# Wave intensity analysis and assessment of myocardial perfusion abnormalities in patients with hypertrophic cardiomyopathy

**DOI:** 10.1186/1532-429X-17-S1-Q72

**Published:** 2015-02-03

**Authors:** Claire E Raphael, Li-Yueh Hsu, Anders M Greve, Robert Cooper, Peter Gatehouse, Ricardo Wage, Vassilis Vassiliou, Aamir Ali, Ranil de Silva, Rodney H Stables, Carlo Di Mario, Kim H Parker, Dudley J Pennell, Andrew E Arai, Sanjay K Prasad

**Affiliations:** 1 Department of CMR, Royal Brompton Hospital, London, UK; 2 National Institutes of Health, Bethseda, MD, USA; 3 Liverpool Heart and Chest Hospital, Liverpool, UK; 4 Bioengineering, Imperial College, London, UK

## Background

Coronary perfusion pressure is a combination of proximal perfusion pressure and distal coronary artery pressure resulting from contraction and relaxation of the myocardium and the consequences on the microcirculation. Wave intensity analysis (WIA) defines dominant waves causing coronary filling during the cardiac cycle and allows separation of proximally and distally originating waves.Reduced myocardial perfusion has been shown to predict mortality in hypertrophic cardiomyopathy (HCM), however no study has assessed the relationship between coronary filling patterns and downstream absolute myocardial perfusion.

## Methods

To obtain data for WIA, simultaneous pressure and coronary flow velocity in the proximal coronary arteries was obtained in 20 patients with HCM at rest and with adenosine. Patients then underwent cardiovascular magnetic resonance (CMR) perfusion imaging at rest and with adenosine. Myocardial blood flow (MBF) was quantified from CMR perfusion images using a pixel-wise model-constrained deconvolution algorithm.

## Results

All patients completed the study successfully. No patients had significant epicardial coronary artery disease. On WIA, distally originating waves predominated compared to proximally occurring waves. The backward expansion wave, which occurs due to myocardial relaxation, correlated with MBFat rest (r=0.66, p=0.01). On CMR, myocardial perfusion reserve was reduced (mean 1.52±0.42) withan endocardial to epicardial ratio that worsened with adenosine (0.96±0.07 at rest vs 0.85±0.11 at stress, p=0.005).

## Conclusions

Patients with HCM had predominantly distal waves governing coronary filling, emphasisingthe importance of the microcirculation in this disease. There was an increased backward compression wave during systole in keeping with compression of the microcirculation with a relative reduction in the size of the backward expansion wave. CMR demonstrated the downstream effects of these abnormalities, with reduced myocardial perfusion and an abnormal endocardial to epicardial ratio. Patients with a smaller backward expansion wave had a lower MBF, suggesting that impaired myocardial relaxation results in poorer myocardial perfusion.

## Funding

This work was funded by the British Heart Foundation and the NIHR Cardiovascular BRU.

